# Corilagin inhibits breast cancer growth via reactive oxygen species‐dependent apoptosis and autophagy

**DOI:** 10.1111/jcmm.13647

**Published:** 2018-06-19

**Authors:** Yinping Tong, Gongye Zhang, Yang Li, Jiajia Xu, Jiahui Yuan, Bing Zhang, Tianhui Hu, Gang Song

**Affiliations:** ^1^ Cancer Research Center Medical College of Xiamen University Xiamen China; ^2^ Fisheries college Jimei University Xiamen China; ^3^ Department of Basic Medicine Medical College of Xiamen University Xiamen China

**Keywords:** breast cancer, corilagin, programmed cell death, reactive oxygen species

## Abstract

Corilagin is a component of *Phyllanthus urinaria* extract and has been found of possessing anti‐inflammatory, anti‐oxidative, and anti‐tumour properties in clinic treatments. However, the underlying mechanisms in anti‐cancer particularly of its induction of cell death in human breast cancer remain undefined. Our research found that corilagin‐induced apoptotic and autophagic cell death depending on reactive oxygen species (ROS) in human breast cancer cell, and it occurred in human breast cancer cell (MCF‐7) only comparing with normal cells. The expression of procaspase‐8, procaspase‐3, PARP, Bcl‐2 and procaspase‐9 was down‐regulated while caspase‐8, cleaved PARP, caspase‐9 and Bax were up‐regulated after corilagin treatment, indicating apoptosis mediated by extrinsic and mitochondrial pathways occurred in MCF‐7 cell. Meanwhile, autophagy mediated by suppressing Akt/mTOR/p70S6K pathway was detected with an increase in autophagic vacuoles and LC3‐II conversion. More significantly, inhibition of autophagy by chloroquine diphosphate salt (CQ) remarkably enhanced apoptosis, while the caspase inhibitor z‐VAD‐fmk failed in affecting autophagy, suggesting that corilagin‐induced autophagy functioned as a survival mechanism in MCF‐7 cells. In addition, corilagin induced intracellular reactive oxygen species (ROS) generation, when reduced by ROS scavenger NAC, apoptosis and autophagy were both down‐regulated. Nevertheless, in SK‐BR3 cell which expressed RIP3, necroptosis inhibitor Nec‐1 could not alleviate cell death induced by corilagin, indicating necroptosis was not triggered. Subcutaneous tumour growth in nude mice was attenuated by corilagin, consisting with the results in vitro. These results imply that corilagin inhibits cancer cell proliferation through inducing apoptosis and autophagy which regulated by ROS release.

## 1 INTRODUCTION

Breast cancer is a primary carcinoma in threatening women's health, the incidence of which is rising in many countries.^1^ In addition to its hereditary, the increasing risk factors are mainly late age of pregnancies, shorter or no periods of breastfeeding caused by pressure of competition and works with the development of economy. Obesity or inactivity caused by bad living habits can also increase the incidence. Although great advances have been made in treating breast cancer through using various therapies, which include chemotherapy, endocrine therapy and radiotherapy,^2^, ^3^ many problems such metastasis, tumour recurrence or drug resistance still exist after receiving treatments above mentioned. Consequently, it is urgent to expand treatments and find more impactful drugs for treating breast cancer.

Corilagin, a polyphenol tannic acid, extracting from *Phyllanthus urinaria*, possesses antiviral, anti‐inflammatory, anti‐oxidant, hypolipemic, hypotensive and anti‐coagulation activities.^4^, ^5^ Many recent studies show that corilagin has effect in tumour therapy as a potential agent candidate. These researches demonstrated that corilagin could control cancer cell growth through regulating various cell signal pathways in different cancers,such as glioblastoma,^6^ hepatocellular carcinoma^7^, ^8^ and ovarian cancer.^9^, ^10^ Corilagin could significantly diminish inflammation through reducing the secretion of IL‐8, MCP‐1 and RANTES in cystic fibrosis bronchial IB3‐1 cells.^11^ Besides, corilagin shows inhibition of cell growth through blocking the activation of both canonical Smad and non‐canonical ERK/AKT pathways in ovarian cancer.^9^ Another study shows that in colitis, corilagin could suppress the secretion of inflammatory cytokines, inhibit the degradation of IκB α,^12^ and besides, it is worth noting that corilagin meanwhile inhibits apoptosis of intestinal epithelial cells.^12^ Despite the fact that corilagin has biological activities in anti‐inflammatory and anti‐tumour, the possibility of its function or even mechanism in inhibiting breast cancer is still uncertain.

Programmed cell death (PCD) plays an important role in the growth and development of multi‐cellular organisms and in regulating homeostasis. Apoptosis, autophagy and necroptosis are the three main forms of PCD, easily distinguished by morphological differences.^13^ Apoptosis was mediated through four main pathways, among them the death receptor‐mediated extrinsic pathway and the intrinsic mitochondrial pathway are two core pathways and intensively studied especially in tumour research.^13^ Caspases,adapter proteins (caspase activators), Bcl‐2 family proteins and inhibitor of apoptosis proteins (IAPs) are main components in participating cascades reactions.^14^ Autophagy is activated under pathological conditions such as nutrient deprivations and unfavourable stress^15^ and is a regulated cell survival process of degradation and recycling of cellular constituents within autophagosome.^16^ Nevertheless, autophagic cell death may be evoked after excessive autophagy.^17^ Necroptosis, or programmed necrosis, belonging to necrosis as a special form, is defined as RIP1/RIP3‐dependent cell death and can be specifically inhibited by an RIP1 inhibitor Nec‐1.^18^, ^19^ Existing studies show that autophagy and apoptosis cooperate to regulate cell survival and death. However, the relationship between autophagy and apoptosis in breast cancer cell is uncertain.

Reactive oxygen species are generally small and short‐lived reactive molecules that are formed by incomplete one‐electron reduction of oxygen.^20^ As natural byproducts of normal metabolism, ROS play important roles in cell signalling and homoeostasis, including autophagy, by modulating theirs level. Studies indicate that salinomycin‐induced ROS may result in abortive autophagy and lead to regulated necrosis in glioblastoma.^21^ Moreover, ROS are proved to be critical in linking autophagy with apoptosis through mitochondria.^22^

In this study, we demonstrated that corilagin simultaneously induced apoptosis and autophagy in MCF‐7 cells, inhibition of autophagy enhanced corilagin‐mediated cell death. Furthermore, cell death introduced by corilagin was proved of relation to ROS formation. Meanwhile, we found corilagin could suppress proliferation but could not induce necroptosis in SK‐BR3 cells. Finally, cell death induced by corilagin in vitro was indeed observed in vivo.

## 2 MATERIALS AND METHODS

### 2.1 Reagents and antibodies

Corilagin (G0424), 3‐(4,5‐Dimethylthiazol‐2‐yl)‐2,5‐diphenyltetrazolium (MTT) (M2128), dimethylsulfoxide (DMSO) (D2650), EBSS (E2888), N‐acetyl‐L‐cysteine (NAC) (A7250) were purchased from Sigma‐Aldrich (St.Louis, MO, USA). Necrostatin‐1 (NEC‐1) (S8037) and z‐VAD‐fmk (Z‐VAD) (S7023) were purchased from Sellck chem and chloroquine (CQ) (4109) was purchased from Tocris. DCFH‐DA (KGT010) was purchased from KeyGBio TECH (China). Lactate dehydrogenase (LDH) (C0016) assay kit was purchased from Beyotime. Caspase‐Glos3(AC030)/8(AC056) Assay Kit were purchased from Promega (Madison, WI, USA).

The following antibodies were used: Caspase‐3 (AC033), caspase‐8 (AC056), caspase‐9 (AC062), Bax (AB026), Bcl2 (AB112) and PARP (AP102) were purchased from Beyotime Biotechnology (Shanghai, China). Anti‐LC3 (NB100‐2220) was purchased from Novus Biological, and anti‐RIP3 (GTX107574) was purchased from Genetex Biological. Phospho‐Akt (81283), Akt (8805), phospho‐mTOR (109268), mTOR (32028), phospho‐p70S6K (126818), p70S6K (32529), phospho‐4EBP1 (75767), 4EBP1 (32024), KI‐67 (15580) and PCNA (29) were obtained from Abcam (Cambridge, MA, USA).

### 2.2 Cell culture

The human breast cancer cell line MCF‐7, SK‐BR3 (obtained from Chinese Academy of Sciences, Shanghai, China) and MDA‐MB‐231 (donated by school of life science of Xiamen University) were cultured in DMEM (Gibco, Grand Island, NY, USA) supplemented with 10% foetal bovine serum (Gibco, Grand Island, NY, USA), 100 U/mL penicillin/streptomycin in an atmosphere of 5% CO2 and 95% air at 37°C. The non‐tumorigenic normal human mammary epithelial MCF‐10A cell, gastric epithelial GES‐1 cell and hepatic epithelia L02 cells were donated by school of life sciences of Xiamen University.

### 2.3 Cell viability assay

Cells were seeded at a density of 5 × 10^3^ cells per well in a 96‐well plate and then treated with corilagin. Added 20 μL MTT (5 mg/mL) for 4 hours and 150 μL DMSO. The absorbance was measured at 490 nm using a plate reader.

### 2.4 EdU proliferation assay

Cell proliferation was detected using the EdU Cell Proliferation Assay Kit (Ruibo, Guangzhou, China).^23^ The number of cells that incorporated EdU was determined by fluorescence microscopy.

### 2.5 Colony formation assay

Cells were seeded in 6‐well plate (500 cells/well) overnight and treated with corilagin. The plates were incubated for another 5 days. Colonies were fixed with methanol, stained with crystal violet, washed with PBS and then counted.

### 2.6 Hoechst 33342 Staining

Cells were fixed with paraformaldehyde, washed and stained with 10 mg/L Hoechst33258 (Promega) for 15 minutes at 37°C. The nuclear morphology was observed under fluorescence microscope.

### 2.7 Lactate dehydrogenase Release assay

Cell death was attested by determining LDH released using a LDH assay kit (Beyotime biotechnology).

### 2.8 Caspase activity measurement

Caspase‐Glos3/8 Assay Kit (Promega) was used for caspase activity measurement following the manufacturer's instructions.

### 2.9 Transmission electron microscopy imaging

Cells were fixed with 2.5% glutaraldehyde in phosphate‐buffered saline, followed by 2% OsO4. After dehydration, thin sections were stained with uranyl acetate and lead citrate for observation under a SEM (JEM‐2100hc, Hitachi, Tokyo, Japan).

### 2.10 Immunofluorescence

Immunofluorescence was performed as in our previous publications.^24^ Cells were fixed and subsequently incubated with anti‐LC3 antibody (1:300) at 4°C overnight, stained with secondary antibody (Boster) (1:300). The image was observed under confocal microscopy.

### 2.11 Flow cytometry

We used propidium iodide (PI, 5 mg/mL) for cell viability analysis and DCFH‐DA (10 μmol/L) for detecting ROS production. The samples were analysed by flow cytometry (Becton Dickinson FACScan).

### 2.12 Western blot analysis

Western blotting was performed as previous described.^25^ In short, samples were collected by lysing cells in RIPA lysis buffer. Each sample was size fractionated using SDS‐polyacrylamide gel electrophoresis (PAGE) and electro transferred onto polyvinylidene difluoride transfer membranes (Dupont, Boston, MA, USA). After bolted with milk, the membranes incubated with primary antibodies overnight at 4°C and then blotted with horseradish peroxidase conjugated secondary antibodies. The immunoblots were visualized using ECL (GE Healthcare, Bucks, UK).

### 2.13 Xenograft assays in nude mice

For xenograft experiments, MCF‐7 cells were implanted by subcutaneous injection into the right foreleg of the female Balb/c nude mice.^26^ A known value of 5, 15, 25 mg/kg of corilagin were intraperitoneally injected every 3 days. The mice were sacrificed after four weeks. Tumour volume was calculated as 1/2ab2.

### 2.14 Statistic analysis

Statistical analyses were performed to compare the differences between the groups using the unpaired Student's *t* test with Prism 5 software. All data are expressed as mean ± standard deviation (SD) or standard error of mean (SEM), and *P* value less than.05 was considered statistically significant.

## 3 RESULTS

### 3.1 Corilagin suppress growth in MCF‐7 cells but not in normal cells

**Figure 1:**
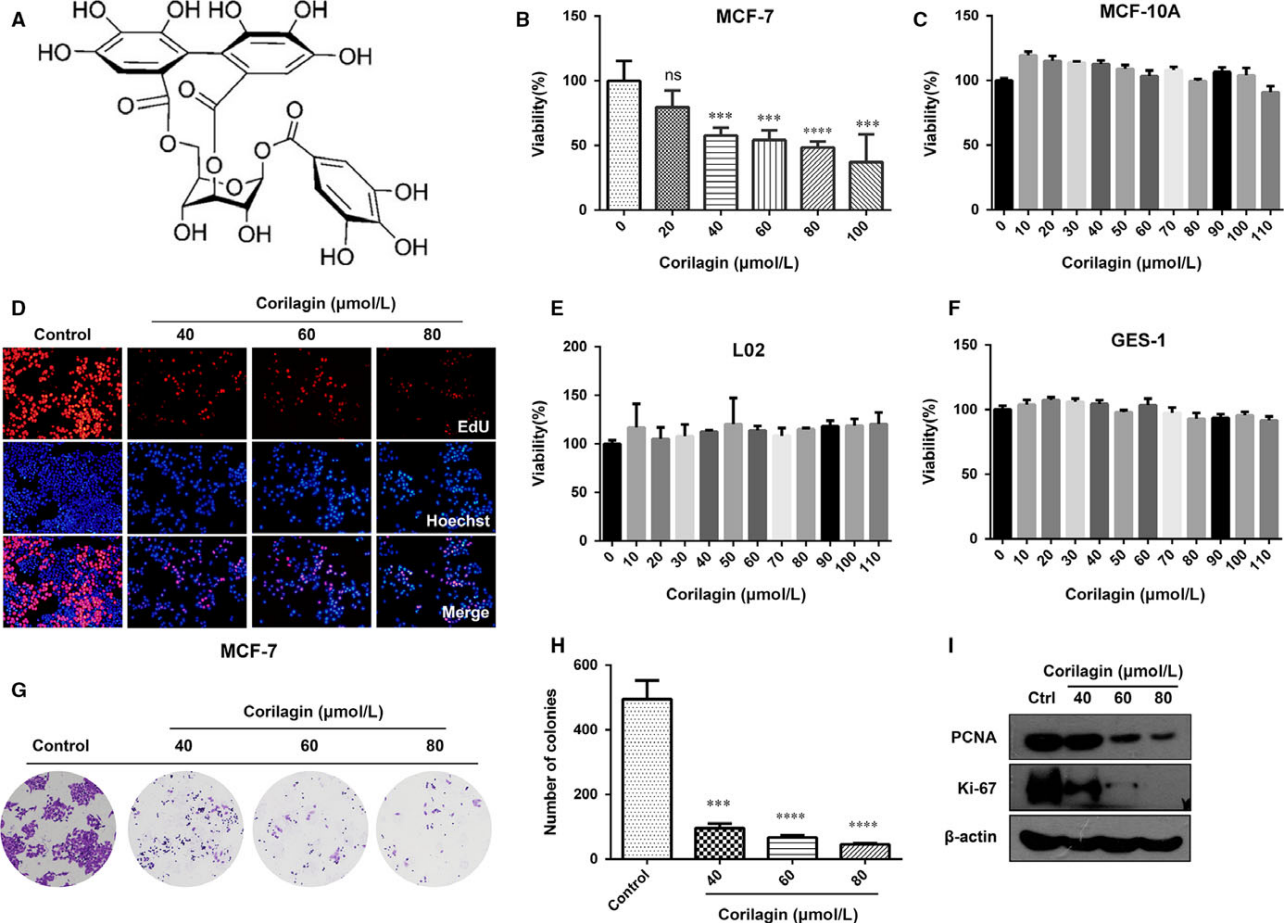
Corilagin suppresses the growth of MCF‐7. (A) The chemical structure of corilagin. (B) MCF‐7 cells were treated with 0, 20, 40, 60, 80, 100 μmol/L corilagin for 48 h, Cell viability were analysed by MTT assay. (C) MCF‐10A, (E) L02, (F) GES‐1 cells were treated with corilagin at concentrations ranging from 0 to 110 μmol/L for 24 h. Cell viability was analysed using MTT assay. (D) EdU assay was performed to assess the growth inhibiting effects to MCF‐7 cells. (G and H) Representative images of colony‐forming assay and its counting results. (I) MCF‐7 cells were treated with different concentrations of corilagin for 24 h. The total protein was extracted, and the expression of PCNA and Ki‐67 proteins was analysed by Western blot assay. Data are expressed as means (n ≥ 3) ± SD over controls, ***P <* *.001, ****P <* *.0001

To investigate the cytotoxic effect of corilagin (structure in Figure 1A) in human breast cancer MCF‐7 cells, MTT and EdU assay were employed. Results showed that corilagin inhibited viability (Figure 1B) and proliferation (Figure 1D) of MCF‐7 cells in a dose‐dependent manner. Additionally, corilagin markedly decreased clonogenicity (Figure 1G and H) and protein expression of PCNA and KI‐67 (Figure 1I), which demonstrated corilagin notably suppress growth in MCF‐7 cells. We also utilized breast cancer cell lines MDA‐MB‐231 and Bcap‐37 to detect the effects of corilagin on them, as they both showed a certain degree of drug resistance (Figure S1Cand D) comparing with MCF‐7, we chose MCF‐7 as our target to further study. Besides, we detected that corilagin had a high efficiency in depressing the viability of colorectal adenocarcinoma cells HT‐29 (Figure S1E) and cervical carcinoma cells Hela (Figure S1F).

In addition, experiments on corilagin‐treated normal cells were performed to investigate whether corilagin has targeting property. MTT assay revealed that cell viability was not decreased in corilagin‐treated mammary epithelial cells MCF‐10A, hepatic epithelial cells L02 and gastric epithelial cells GES‐1(Figure 1C, E and F). Besides, EdU assay showed that corilagin treatment group had no difference with control group in GES‐1 cells (Figure S1A) and L02 cells (Figure S1B). These data demonstrate that corilagin can specifically inhibit the growth of breast cancer cells MCF‐7 and barely suppress normal cells.

### 3.2 Corilagin activate extrinsic and intrinsic mitochondrial apoptosis pathways in MCF‐7 cells

**Figure 2:**
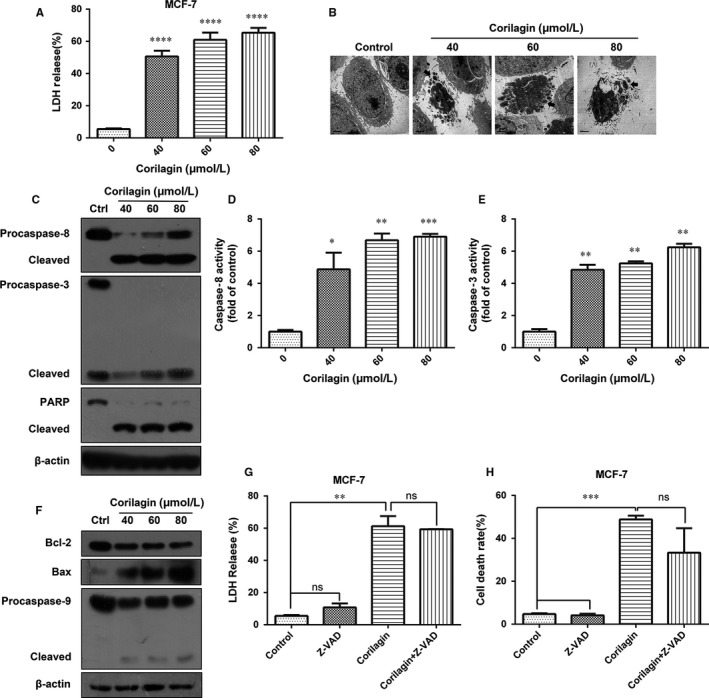
Corilagin introduces apoptosis in MCF‐7. (A) MCF‐7 cells were incubated for 24 h with 0, 40, 60, 80 μmol/L corilagin. Cell death was analysed with the LDH release assay. (B) Transmission electron microscopic observation was performed on MCF‐7 cells treated with corilagin for 24 h to detect apoptosis. (C) MCF‐7 cells were treated with different concentrations of corilagin for 24 h. The total protein was extracted and the expression of caspase‐3, caspase‐8, caspase‐9, PARP and the cleaved forms were analysed by Western blot assay. MCF‐7 cells were treated with corilagin for 24 h. Caspase activities were determined with colorimetric assays using (D) caspase‐8 IETDase assay kits and (E) caspase‐3 DEVDase assay kits. (F) MCF‐7 cells were treated with different concentrations of corilagin for 24 h. The total protein was extracted, and the expression of Bax, Bcl‐2, procaspase‐9 and cleaved form was analysed by Western blot assay. (G) MCF‐7 cells were pretreated with z‐VAD‐fmk (20 μmol/L) for 2 h, then corilagin (60 μmol/L) treatment for 24 h. z‐VAD‐fmk efficiency was analysed by the LDH release assay. (H) MCF‐7 cells were pretreated with z‐VAD‐fmk (20 μmol/L) for 2 h, then corilagin (60 μmol/L) treatment for 24 h and then dyed with PI. Cell death rate was analysed by flow cytometry. Data are expressed as means (n ≥ 3) ± SD over controls, *P <* *.05, **P <* *.01, ***P <* *.001, ****P <* *.0001. ns: no significant. Abbreviations: LDH, lactate dehydrogenase; PARP, poly(ADP‐ribose) polymerase; PI, propidium iodide

Research showed that corilagin treatment activated apoptosis in ovarian cancer cells, which significantly increased the number of apoptotic cells.^9^, ^10^, ^27^ Then we tried to reveal the mode of cell death induced by corilagin treatment in MCF‐7 cells. LDH release assay showed that the release of LDH increased markedly in corilagin‐treated MCF‐7 cells (Figure 2A), suggesting that cell damage and cell death occurred. Besides, formation of apoptosis body was found by transmission electron microscope (TEM) imaging in corilagin‐treated MCF‐7 cells (Figure 2B), indicating apoptosis was activated.

Corilagin induced apoptosis may be triggered either by extrinsic stimuli or by intrinsic stimuli via the mitochondrial signalling pathway. In either case, activation of caspase leads to destruction of cell.^28^ To investigate whether caspase activation was involved in corilagin‐induced cell death, Western blot was performed and as shown in the results, corilagin treatment markedly increased the levels of the cleaved caspase‐8 and cleaved PARP, while decreased the levels of procaspase‐8, procaspase3 and PARP (Figure 2C). Additionally, activity of caspase‐8 and caspase‐3 was significantly improved (Figure 2D and E). Cell death can also happen through an alternative mitochondrial pathway, detection of the related proteins (Figure 2F) showed that corilagin up‐regulated the expression of Bax and down‐regulated of Bcl2 and procaspase‐9, indicating an intrinsic mitochondrial apoptosis pathway was activated apart from extrinsic pathway. To verify whether caspase‐independent cell death occurred, a pan caspase inhibitor z‐VAD‐fmk was used and found that z‐VAD‐fmk had slight effect on corilagin‐induced cell damage (Figure 2G) and reduction in cell viability (Figure 2H), suggesting the existence of caspase‐independent mechanism. Thus, corilagin induced cell death through both caspase‐dependent and caspase‐independent pathway in MCF‐7 cells.

### 3.3 Corilagin activate autophagy in MCF‐7 cells and autophagy inhibition enhance apoptosis

**Figure 3:**
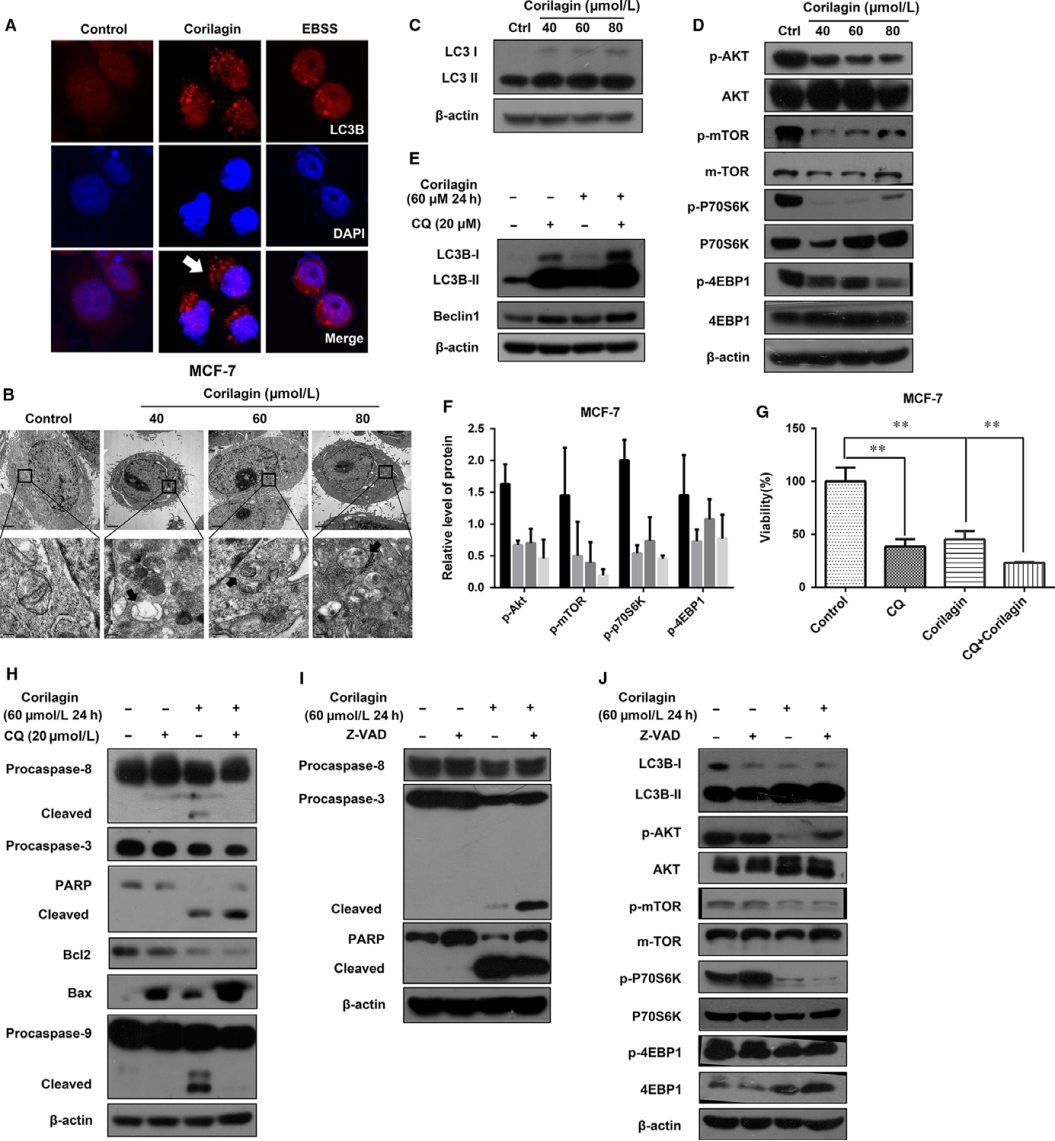
Corilagin activate autophagy in MCF‐7 cells, and autophagy inhibition enhances apoptosis. (A) Cells were treated with corilagin for various concentrations for 24 h, stained with LC3 antibody (Red) and appropriate secondary antibody and observed under a fluorescence microscope, the nuclear was stained with DAPI (Blue). EBSS as a positive control, and LC3 puncta (white arrows) was indicated. (B) Cells were treated as described above. After treatment, cells were harvested and subjected to transmission electron microscopy as described in 2. Autophagosomes with typical double‐layer membranes containing organelle remnants were pointed out by black arrows. (C) Cells were treated with corilagin for various concentrations for 24 h; then, Western blot analysis was performed to detect the expression of LC3. (D) Cells were treated with corilagin for various concentrations for 24 h, and the levels of p‐Akt (Ser473), p‐mTOR(Ser2448), p‐p70S6K(Thr389), p‐4EBP1(Thr37/46), Akt, mTOR, p70S6K and 4EBP1 were detected by Western blot. β ‐actin was used as loading control. (E) Detection of the efficiency of CQ. Cells were pretreated with 20 μmol/L CQ for 2 h and then treated with 60 μmol/L corilagin for 24 h. The level of LC3 and Beclin1 was examined by Western blot analysis. (F) Relative level of proteins in figure D. (G) Cells were pretreated with 20 μmol/L CQ for 2 h and then treated with 60 μmol/L corilagin for 24 h. The level of caspase‐8, caspase‐3, PARP, Bcl2, Bax, and caspase‐9 was examined by Western blot analysis. β‐actin was used as a loading control. (H) Detection of the efficiency of z‐VAD‐fmk. Cells were pretreated with 20 μmol/L z‐VAD‐fmk for 2 h and then treated with 60 μmol/L corilagin for 24 h. The protein levels of caspase‐8, caspase‐3 and PARP were detected by Western blot analysis. (I) Cells were pretreated with 20 μmol/L z‐VAD‐fmk for 2 h and then treated with 60 μmol/L corilagin for 24 h. The level of LC3, Akt, mTOR and the phosphorylation of Akt, mTOR, p70S6K and 4EBP1 was examined by Western blot analysis. β‐actin was used as a loading control. (J) Cell was pretreated with 20 μmol/L CQ for 2 h and then treated with 60 μmol/L corilagin for 24 h. Cell viability was assessed by performing MTT assay. Data are expressed as means (n ≥ 3) ± SD over controls, **P <* *.01. Abbreviations: EBSS, Earle's Balanced Salts; CQ, chloroquine

To ascertain whether corilagin activate autophagy, immunofluorescence was applied to verify the morphologic change of the corilagin‐treated MCF‐7 cells, and autophagosomes were detected (Figure 3A). To further confirm autophagy, we checked detailed cellular images with TEM and observed an extensive intracellular autolysosome‐like vesicles and cellular debris (Figure 3B). Then we checked LC3 conversion by Western blot and found that conversion of LC3 exhibited on account of corilagin treatment (Figure 3C). Results revealed that corilagin induced the formation of LC3 puncta and autophagosomes. These findings indicated that corilagin induced autophagic cell death.

To better apprehend the underlying mechanisms of corilagin‐induced autophagy, we detected the Akt/mTOR pathway, which was closely related to autophagy.^29^ Corilagin dramatically down‐regulated the phosphorylation of Akt and mTOR, as well as that of substrates p70S6kinase and 4EBP1 (Figure 3D and F), indicating that corilagin induce autophagy through Akt/mTOR/p70S6K pathway.

Several studies revealed autophagy may act as a protective mechanism in tumour cells and autophagy inhibition could augment apoptosis.^30^ To test whether autophagy protect cancer cell from death, we used chloroquine (CQ), an inhibitor for late autophagy, and corilagin to coprocessing MCF‐7 cells. The conversion of LC3 and viability was then investigated by Western blot and MTT assay, respectively. The results showed that corilagin co‐treatment with CQ increased the level of LC3B‐II, Beclin1 (Figure 3E) and further suppressed cell viability (Figure 3G) compared with corilagin‐treated alone.

To further confirm that the decrease in viability was evoked by apoptosis after CQ co‐exposure, we examined expression levels of apoptosis‐related proteins by Western blot. Corilagin co‐treatment with CQ further decreased the level of procaspase‐3, PARP and Bcl‐2, and increased cleaved caspase‐8, cleaved PARP, Bax and cleaved caspase‐9 comparing with corilagin treatment alone (Figure 3H). These data indicated that blocking autophagy enhanced corilagin‐induced apoptosis.

To check the interplay between autophagy and apoptosis, the effect of apoptosis inhibition on autophagy was investigated. We first examined the effect of z‐VAD‐fmk by detecting apoptosis‐related proteins in corilagin and z‐VAD‐fmk pretreated MCF‐7 cells (Figure 3I), indicating that z‐VAD‐fmk was effective. To detect whether autophagy also had a change, we tested LC3 and Akt/mTOR pathway‐related protein. The results showed that z‐VAD‐fmk had no effect on autophagy (Figure 3J). Thus, we concluded that corilagin‐induced autophagy which inhibited apoptosis may act as a protective role in MCF‐7 cells.

### 3.4 Autophagy and apoptosis were correlated with the production of ROS in MCF‐7 cells

**Figure 4:**
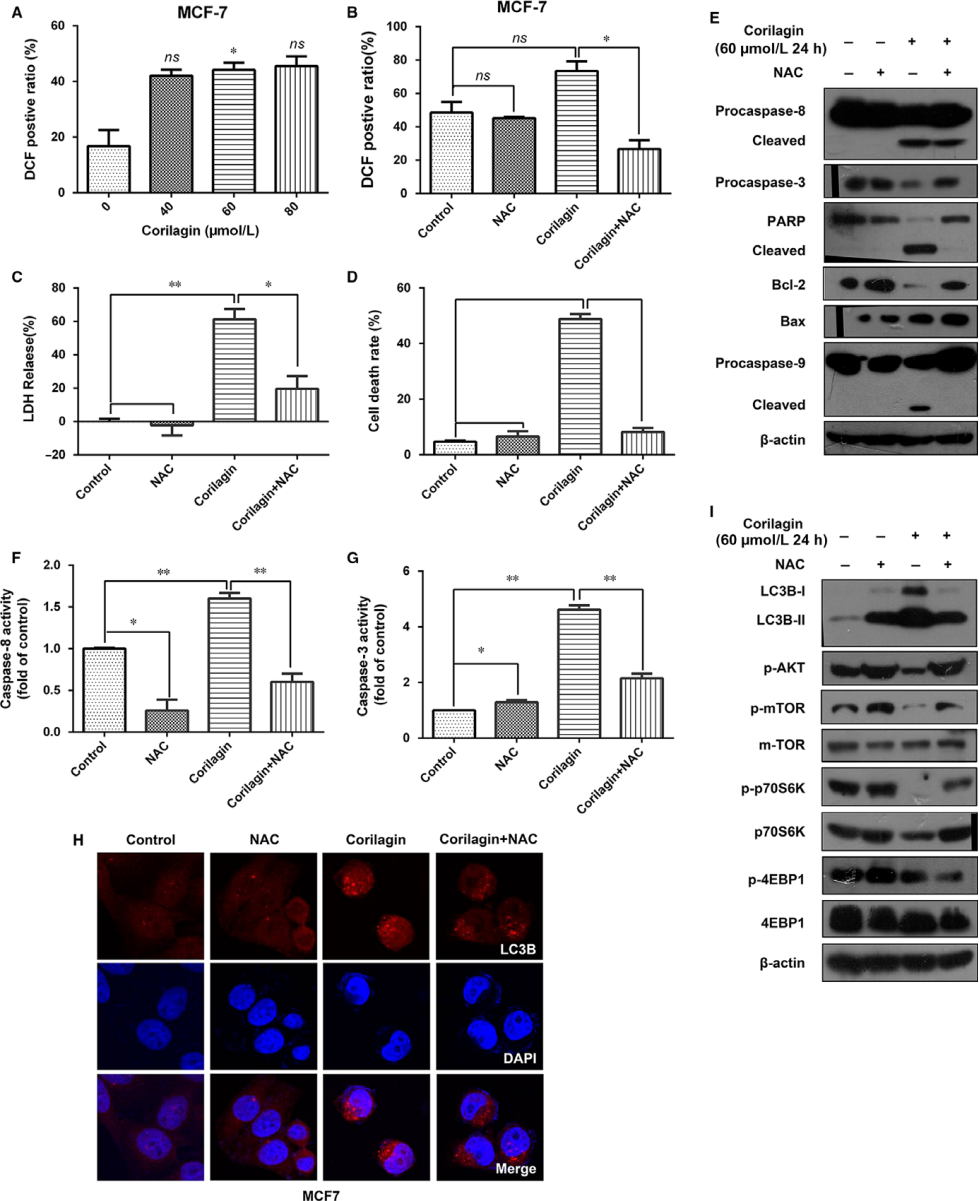
Influences of ROS to autophagy and apoptosis in corilagin treated MCF‐7 cells. (A) MCF‐7 cells were incubated with corilagin at the different concentrations for 24 h and then stained with H2DCF‐DA (10 μmol/L). ROS level was analysed by flow cytometry. (B) Detection of the efficiency of NAC. MCF‐7 cells were pretreated with NAC (5 mmol/L) for 2 h and then corilagin (60 μmol/L) treatment for 24 h, stained with H2DCF‐DA (10 μmol/L). ROS level was analysed by flow cytometry. (C) Detection of the influence of NAC to cell damage. MCF‐7 cells were pretreated with NAC (5 mmol/L) for 2 h and then corilagin (60 μmol/L) treatment for 24 h. Cell death was analysed with the LDH release assay. (D) MCF‐7 cells were pretreated with NAC (5 mmol/L) for 2 h and then corilagin (60 μmol/L) treatment for 24 h. Cells were stained with propidium iodide (PI) and then analysed by flow cytometry to detect cell death rate. (E) Cells were pretreated with 5 mmol/L NAC for 2 h and then treated with 60 μmol/L corilagin for 24 h. The protein levels of Bcl2, Bax, caspase‐8, caspase‐3, PARP, Bcl2, Bax, and caspase‐9 were detected by Western blot analysis. (F) Caspase‐8 activities and (G) caspase‐3 activities were determined using caspase‐8 IETDase assay kits and caspase‐3 DEVDase assay kits, respectively. (H) Confocal images present LC3B (Red) expression of autophagosome formation in MCF‐7 cells treated with vehicle, NAC, corilagin, corilagin plus NAC. (I) Western blots show the effects of NAC on corilagin induced LC3II conversion and the protein levels of mTOR and the phosphorylation of Akt, mTOR, p70S6K and 4EBP1 in MCF‐7 cells. Data are expressed as means (n ≥ 3) ± SD over controls, *p <* *.05, **P <* *.01, ns: no significant. Abbreviations: NAC, N‐acetyl cysteine

Reactive oxygen species have important roles in intracellular signal transduction and redox homoeostasis.^31^ In our model, we observed that corilagin increased intracellular ROS generation (Figure 4A). To further reveal the role of ROS, we blocked production of ROS using ROS scavenger N‐acetylcysteine (NAC) (Figure 4B), and NAC shows no damage to cells (Figure 4C). We examined cell viability by MTT assay, and the results revealed NAC completely relieved corilagin's effect on cell viability (Figure 4D).

To further investigate whether ROS play a key role in corilagin‐induced apoptosis and autophagy. On the one hand, we checked the changes of apoptosis‐related protein. Corilagin and NAC co‐treatment significantly decreased cleaved caspase‐8, caspase‐9, PARP and increased Bcl‐2 compared with corilagin treated alone (Figure 4E). Additionally, caspase‐8 and caspase‐3 activities were suppressed (Figure 4F and G). On the other, to detect the effect on autophagy, we examined the expression changes of autophagy‐related protein. Results displayed that LC3 puncta was inhibited (Figure 4H), and the level of LC3II along with the proteins such as p‐mTOR and p‐p70S6K related to Akt/mTOR pathway (Figure 4I) was restored after NAC treatment. Taken together, ROS had influence on corilagin‐induced cell death and maybe participate in regulating autophagy and apoptosis in MCF‐7 cells. Besides, the increase in the level of autophagy induced by NAC treatment was consistent with the conclusion aforementioned that autophagy protected MCF‐7 cells from death.

### 3.5 Corilagin cannot induce necroptosis in SK‐BR3 cells

Recently, programmed necrosis is found in cancer cell death by anticancer agents.^29^, ^32^ Activation of the canonical programmed necrosis includes the formation of a complex containing RIP3 and RIP1 (RIPK1) and recruitment of mixed lineage kinase domain‐like protein (MLKL).^33^, ^34^

**Figure 5:**
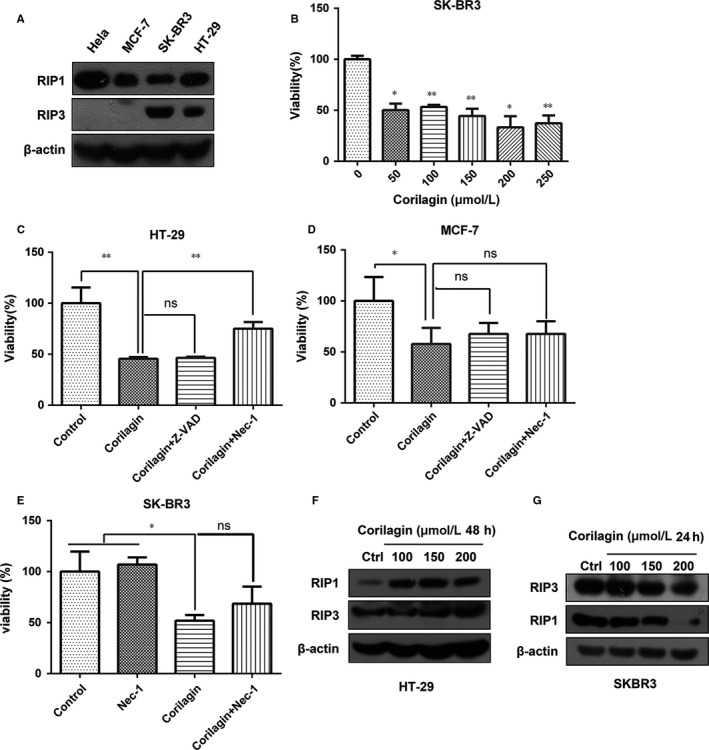
Corilagin cannot induce necroptosis in SK‐BR3 cells. (A) The protein levels of RIP1 and RIP3 were detected by Western blot analysis in different cell lines. (B) SKBR3 cells were treated with 0, 50, 100, 150, 200, 250 μmol/L corilagin for 48 h. MTT assay was performed to assess the growth inhibiting effects. (C) HT‐29 cells and (D) MCF‐7 cells were pretreated with z‐VAD‐fmk and Nec‐1 for 2 h and then co‐treatment with corilagin for 24 h. Cell viability was detected by MTT assay. (E) SK‐BR3 cells pretreated with Nec‐1 for 2 h and then co‐treatment with 100 μmol/L corilagin for 48 h. Cell viability was detected by MTT assay. (F) HT‐29 cells and (G) SK‐BR3 cells were treated by corilagin. The expression of RIP3 and RIP1 was detected by Western blot. Data are expressed as means (n ≥ 3) ± SD over controls, *P <* *.05, **P <* *.01, ns: no significant. Abbreviations: Nec‐1, necrostatin‐1

Owing to necroptosis depend on RIP3, we used HT‐29 cells as positive control and Hela cells as negative control, detecting the level of RIP1 and RIP3 in MCF‐7 and SK‐BR3, and found that the level of RIP1 was similar, while SK‐BR3 cells expressed RIP3 and MCF‐7 not (Figure 5A). Then we chose SK‐BR3 as object to investigate the cytotoxic effect of corilagin in human breast cancer. MTT assay showed proliferation was inhibited in corilagin treatment SK‐BR3 cells (Figure 5B).

To clarify whether corilagin induced necroptosis in SK‐BR3 cells, we used Nec‐1, an inhibitor for RIP1‐dependent programmed necrosis, to detect whether cell viability affected by Nec‐1. Results indicated that Nec‐1 markedly restored cell viability in corilagin‐treated HT‐29 cells (Figure 5C), but not in MCF‐7 and SKBR3 cells (Figure 5D and E). To further confirm the data, we performed Western blot to detect whether the level of RIP1 and RIP3 was increased. Results showed that corilagin up‐regulated expression of RIP1, RIP3 in HT‐29 cells (Figure 5F). However, RIP1 and RIP3 were decreased in SK‐BR3 cells (Figure 5G, Figure S2). These results demonstrated that corilagin cannot induce necroptosis in breast cancer SK‐BR3 cells.

### 3.6 Corilagin inhibits the growth of MCF‐7 xenograft tumours

**Figure 6:**
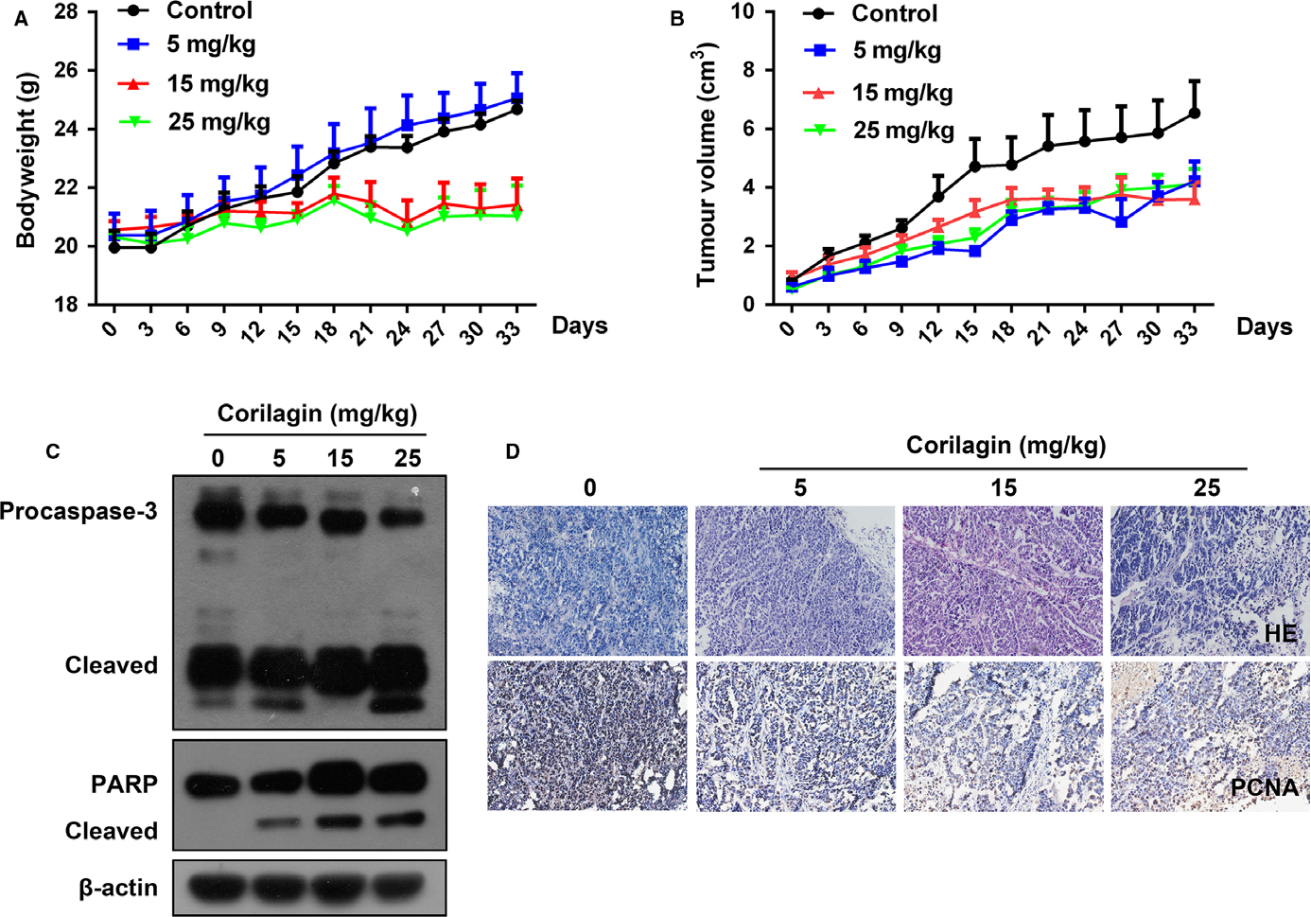
Corilagin inhibits the growth of MCF‐7 xenograft tumours. (A and B) The statistical results of mice weight and tumour volume. (C) The protein levels of caspase‐3, PARP in tumours were detected by Western blot. (D) H&E staining and Immunohistochemical analysis about expression of PCNA in tumour tissue. Data are reported as means (n ≥ 3) with SEM of three separate experiments

It is reported that corilagin had anti‐tumour activity on hepatocellular carcinoma^35^ and cholangiocarcinoma^36^ in vivo. In order to ascertain whether corilagin possess any anti‐tumour effects on breast cancer in vivo, xenograft model was established via injecting MCF‐7 cells subcutaneously into nude mice. Tumour‐bearing animals were randomly assigned to four groups to receive intraperitoneally injection of saline (negative control), 5, 15, 25 mg/kg of corilagin for 4 weeks. As shown in Figure 6A and B, corilagin prevented tumour growth and the mean volume of tumour and bodyweight of mice were obviously lower than the control group. In order to clarify whether corilagin induced apoptosis in vivo, the expression levels of caspase‐3, PARP were tested. Comparing with control group, caspase‐3 and cleaved PARP increased evidently in corilagin‐treated groups (Figure 6C). As shown in Figure 6D, HE staining indicated greater cancer areas in control group. Besides, the PCNA‐positive cells which exhibited brown punctate granules in the nucleus showed higher expression in control group. These results revealed that corilagin can suppress tumour growth in vivo.

## 4 DISCUSSION

As a kind of gallotannin, corilagin has already identified in several plants and is identified as the major active component of *Phyllanthus niruri L*. It has been known that corilagin has effect in hepatoprotective, antiviral, antibacterial and tumour therapy. Studies found that corilagin may control cholangiocarcinoma cell growth by down‐regulating the expression of Notch1,^36^ arrests SMMC7721 cells at the G2/M phase by down‐regulating p‐Akt and cyclinB1/cdc2 and up‐regulating p‐p53 and p21Cip1.^8^ However, few reports examined the concrete mechanism in corilagin mediating cell death. In our study, we first revealed that corilagin induces both ROS‐mediated apoptosis and autophagy in breast cancer MCF‐7 cells, but induces no necroptosis in breast cancer SK‐BR3 cells. Moreover, our research revealed that corilagin induced apoptosis (Figure S3 andB) and autophagy (Figure S3C‐E) in breast cancer MDA‐MB‐231 cells.

Performed cell and animal experiments demonstrated that corilagin suppressed the proliferation of breast cancer cells in vitro and in vivo. The experiment in vivo demonstrated that corilagin inhibited tumour growth without obviously toxicity which reflected on the loss of bodyweight. It should be noted that corilagin has hardly any obvious cytotoxic effect on normal cells, for instance, mammary epithelial MCF‐10A cells, gastric epithelial GES‐1 cells and hepatic epithelia L02 cells (Figure 1). These results imply that corilagin demonstrated better anti‐tumour potential and lower toxicity in normal cells.

Although the fact corilagin can induce apoptosis in many kinds of cancer has been proved in many research, the specific mechanism is not so clear. In our study, we observed that the activity of caspase‐8, caspase‐3 and cleaved caspase‐8, caspase‐9, PARP was up‐regulated after corilagin treatment, inferring that corilagin induced caspase‐dependent apoptosis in MCF‐7 cells. Meanwhile, corilagin up‐regulated the expression of Bax and down‐regulated of Bcl‐2, means intrinsic mitochondrial apoptosis pathway was activated either. Furthermore, we checked whether caspase‐independent pathway is also involved. As expected, we found that z‐VAD‐fmk, a pan inhibitor of caspase, did not markedly block cell death upon corilagin treatment in MCF‐7 cells, indicating that instead of caspase‐dependent pathway, another way of cell death exists, namely caspase‐independent pathway. Taken together, corilagin induced caspase‐dependent and caspase‐independent apoptosis in MCF‐7 cells.

In cancer therapy, autophagy seems to be playing a conflicting role, probably promoting or inhibiting cancer cell survival. The role of autophagy in different therapies is cell line and stimulus dependent.^37^ LC3‐II plays an indispensable role in autophagosomes formation.^38^ By affirming LC3II conversion and formation of autophagosomes, our present results first illustrated that corilagin can evoke autophagy in breast cancer cells. Further investigations were warranted to elucidate the mechanism underlying autophagy induced by corilagin. Previous research has been shown that AMPK activation inhibits the rapamycin (mTOR) signalling pathway.^39^, ^40^ Depending on the binding partners and sensitivities to rapamycin, mTOR resides in at least two distinct complexes, termed mTOR complex 1 (mTORC1) and mTOR complex 2 (mTORC2).^41^ Among the multiprotein components, Raptor (regulatory associated protein of mTOR, complex (i) and Rictor (Raptor independent companion of mTOR, complex (ii) are characteristic components of mTORC1 and mTORC2, respectively.^42^ Most studies targeting the mTOR pathway in cancer therapy mainly focus on rapamycin and its derivatives, which are inhibitors of mTORC1 but not of mTORC2.^43^, ^44^ mTORC1 is well‐known to inhibit autophagy by phosphorylating p70S6K and 4EBP1, and mTORC2 is related to cytoskeleton construction and cell movement. The main factors regulating cell proliferation, apoptosis and autophagy are mTORC1^45^. In our study, we certified that the activation of autophagy was triggered by corilagin via inhibition of Akt/mTOR pathway. Whereas autophagy induction may also be mediated by other factors or not only depend on Akt/mTOR signalling, which are still need to be further examined.

Some recent data indicated there is a link between the apoptosis and autophagy.^46^ Meanwhile, it should be noted that inhibition of autophagy induces apoptosis.^47^ Numerous evidences suggested that autophagy was a survival mechanism which provided energy during metabolic stress and could protect cancer cells from apoptosis or necrosis after various anticancer treatments.^48^, ^49^ Here, we adopted the autophagy inhibitor CQ and the apoptosis suppressor z‐VAD‐fmk. Consistent with these findings, in our research, inhibition of autophagy, particularly at a late stage, facilitated cancer cells to apoptosis in corilagin‐treated MCF‐7 cells. Nevertheless, no changes were observed after z‐VAD‐fmk treatment on autophagy. Besides, it is found that chloroquine has antitumour and anti‐metastatic activities in a murine model of breast cancer.^50^ These data provide a new clue to understand that combined corilagin and CQ treatment may be a promising therapeutic strategy. Nevertheless, the exact mechanisms underlying the pro‐apoptotic action of autophagy in corilagin exposed cancer cells remain unclear. Therefore, further study should be performed to explore the mechanism of the relationship between apoptosis and autophagy. Moreover, the safety and efficacy of the combination of corilagin and CQ therapy should be examined in vivo studies.

ROS are critical signalling molecules. ROS‐mediated apoptosis and autophagy have been observed in various cancer cell types.^51^ In the present study, in virtue of corilagin markedly increased intracellular ROS generation in MCF‐7 cells, and addition of NAC could significantly decrease ROS generation and also markedly restrain cell death. Hence, we suspected corilagin probably activated apoptosis and autophagy through inducing ROS generation. As expected, NAC pretreatment protected MCF‐7 cells from corilagin induced cell apoptosis, including recover the level of caspase. Furthermore, we also found that NAC obviously reduced LC3II expression, restored Akt/mTOR pathway. Therefore, our results suggest that ROS may play a crucial role in corilagin‐induced apoptosis and autophagy in MCF‐7 cells.

Recently, programmed necrosis, namely necroptosis, is found as a new kind of programmed cell death in cancer cell death which was resulted by anticancer agents.^32^ Receptor‐interacting protein kinase‐3 (RIP3 or RIPK3) is an essential part of the cellular machinery that executes the programmed necrotic cell death.^52^, ^53^, ^54^ In our study, we utilized SK‐BR3 cells which express high level of RIP3 to confirm whether necroptosis can be induced by corilagin. Meanwhile, we applied HT‐29 cells as positive control. As expected, corilagin increased the level of RIP3 as well as RIP1, and employing Necrostatin‐1 (Nec‐1), a necrotizing apoptosis inhibitor which could competitively inhibit the activity of RIP1 kinase, can recover cell viability in HT‐29 cells. However, the results displayed that corilagin cannot augment the level of RIP3 as well as RIP1, and Nec‐1 cannot restore cell viability in SKBR3 cells. Therefore, we speculated that corilagin cannot induce necroptosis in breast cancer cells, but further research is needed to verify this conclusion.

Collectively, our research proved orilagin could inhibit breast cancer growth via ROS‐dependent apoptosis and autophagy, and we consider that corilagin is a new promising candidate as a potential antitumour drug for treating breast cancer. In consideration of its high dose, further research could be performed on the area of reducing dose and enhancing effectiveness through modifying structure of corilagin.

## CONFLICT OF INTEREST

The authors declare no conflict of interests.

## AUTHOR CONTRIBUTIONS

YP and GY performed conception and design, experiments performing, data analysis; YL, JJ and JH contributed to experiments performing; BZ and TH performed data analysis. GS contributed to conception and design, data analysis, manuscript writing, financial support, and final approval of manuscript.
