# Supplementary figures and images for: Corilagin inhibits breast cancer growth via reactive oxygen species‐dependent apoptosis and autophagy
Source: J Cell Mol Med. 2018 Jun 19;22(8):3795–807. doi: 10.1111/jcmm.13647 (PMC6050496; doi:10.1111/jcmm.13647)

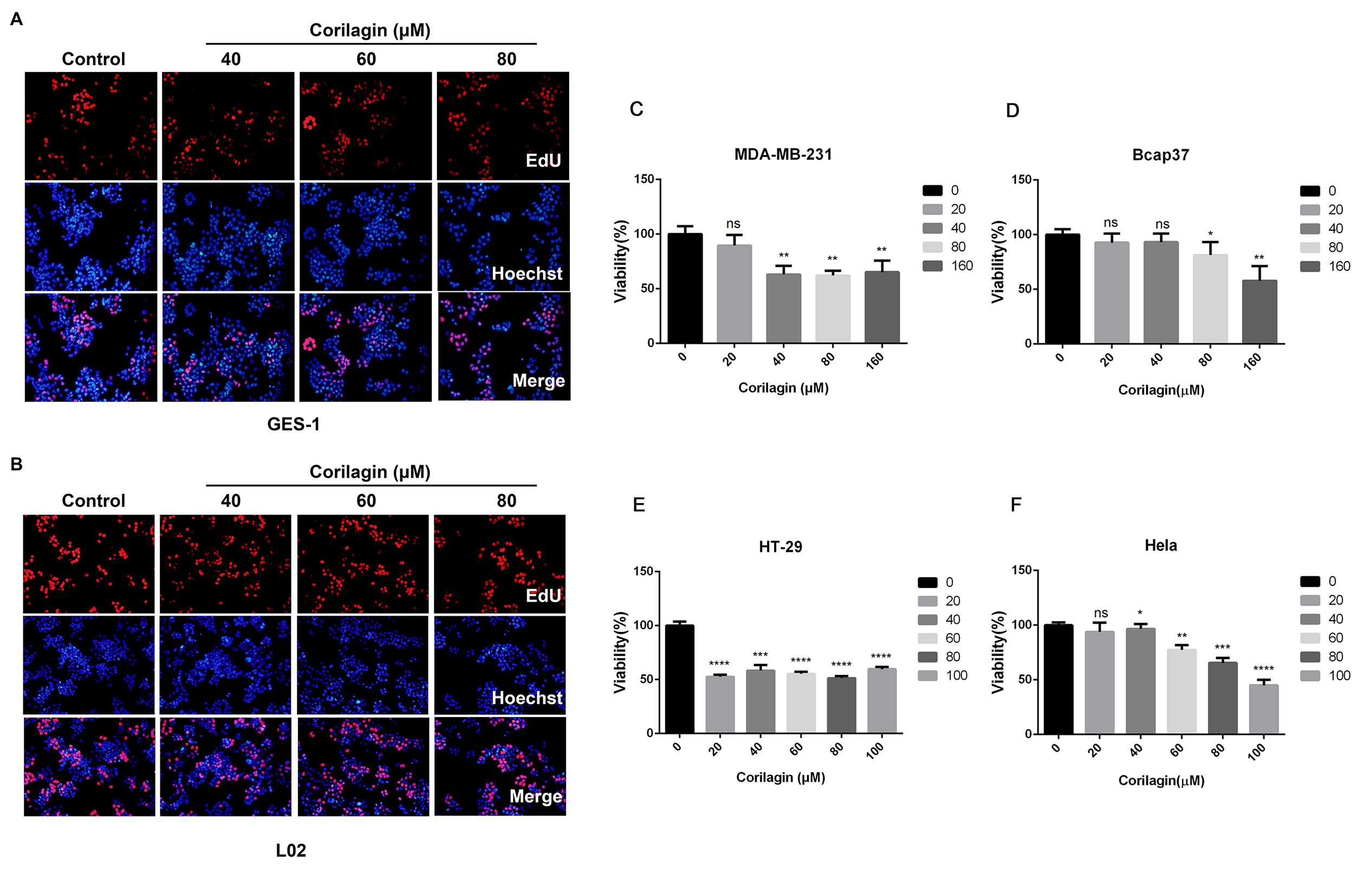

Supplement: Supplementary file 1 [file JCMM-22-3795-s001.tif]

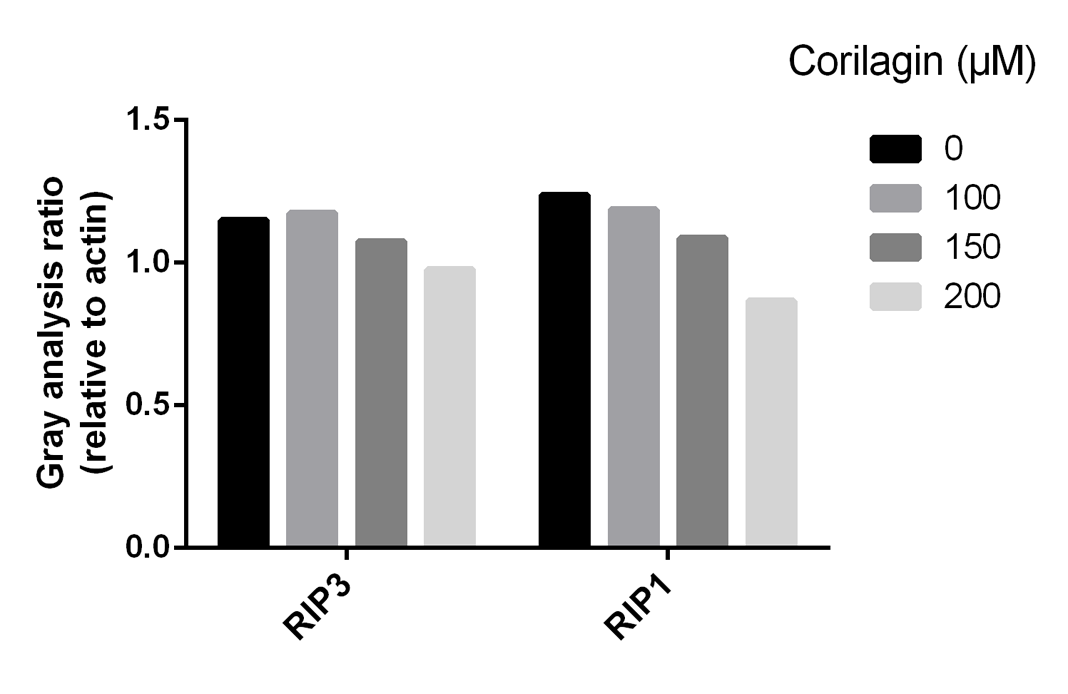

Supplement: Supplementary file 2 [file JCMM-22-3795-s002.tif]

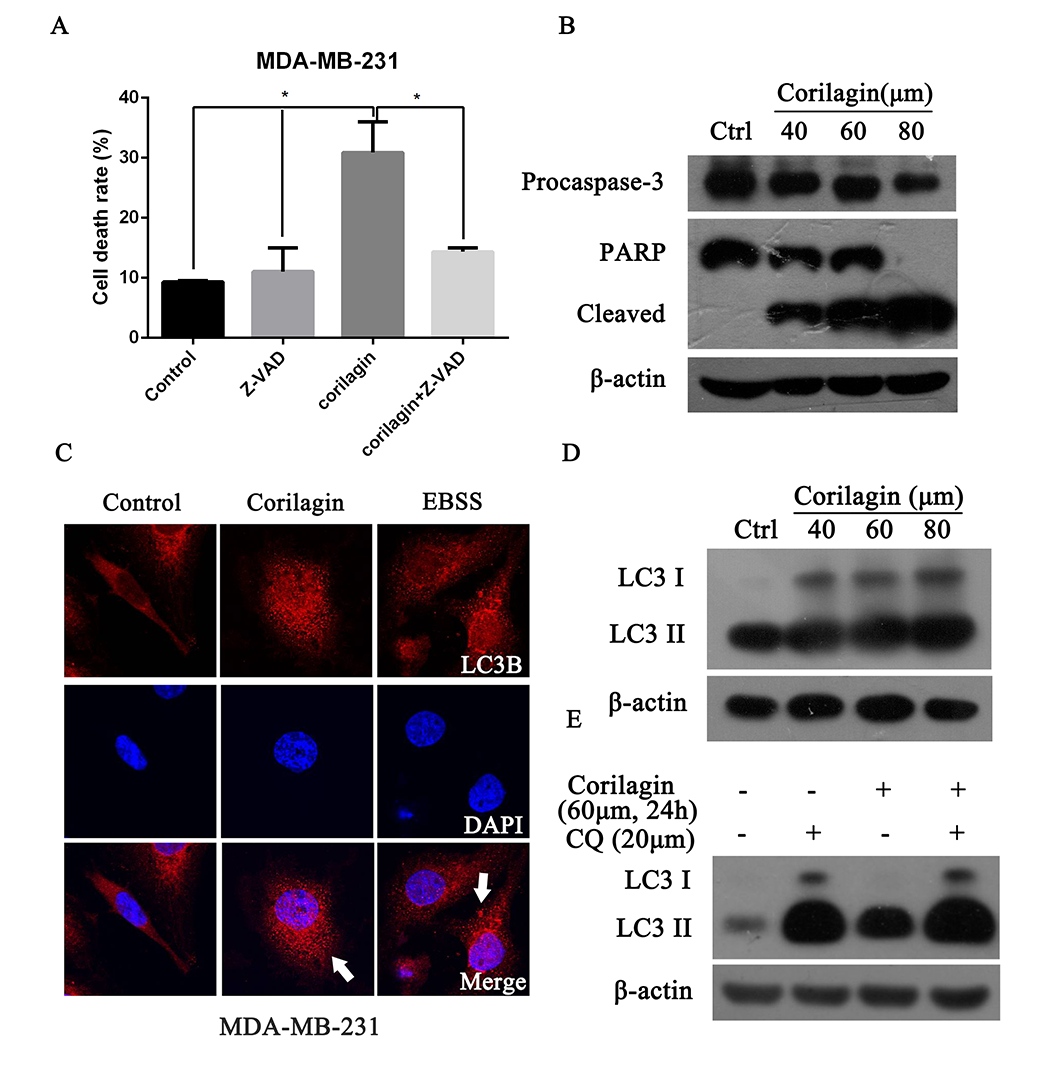

Supplement: Supplementary file 3 [file JCMM-22-3795-s003.tif]
